# SINGLE-TIE CORE NETWORKS AMONG OLDER EUROPEANS: A POSITION OF PRECARITY AND LONELINESS?

**DOI:** 10.1093/geroni/igad104.1382

**Published:** 2023-12-21

**Authors:** Haosen Sun, Jina Lee, Markus Schafer

**Affiliations:** UNR School of Public Health, Reno, Nevada, United States; University of Toronto, Toronto, Ontario, Canada; Baylor University, Waco, Texas, United States

## Abstract

The problems of late-life social isolation and loneliness prompt significant concern. Individuals who have core personal networks limited to a single connection may be especially susceptible to loneliness, particularly when that connection is no longer available. The present research considers: 1) how prevalent such single-tie networks are and who is most likely to embed in them; 2) whether older adults in single-tie networks are more lonely than people with more expanded network forms; and 3) whether such networks put people at highest of loneliness in the event of network member loss. Using ego-centric network data from Waves 4 (2011) and Wave 6 (2015) of the Survey of Health, Ageing, and Retirement in Europe (SHARE), we conduct lagged dependent variable logistic regressions. Results show that a total of 28.2% of older Europeans rely on a single person as an important personal tie. Among those maintaining a single-tie network, spouses are the most common choice (62.4%), followed by a child (15.2%), a relative (8.5%), and a non-relative (13.8%). Factors significantly associated with holding different types of single-tie networks include age, gender, education, financial pressure, rural residence, participation in social activities, and grandparental roles. Child-only networks are significantly associated with greater levels of loneliness compared to multi-tie networks. Meanwhile, the loss of a partner as the only connection is associated with significantly increased loneliness, even after considering possible network additions. Future research should investigate how to better protect older adults in precarious network settings, especially during network losses.

